# Nanoparticles Formulation Improves the Antifibrogenic Effect of Quercetin on an Adenine-Induced Model of Chronic Kidney Disease

**DOI:** 10.3390/ijms23105392

**Published:** 2022-05-12

**Authors:** Esteban Andrés Sánchez-Jaramillo, Luz Elena Gasca-Lozano, José María Vera-Cruz, Luis Daniel Hernández-Ortega, Carmen Magdalena Gurrola-Díaz, Blanca Estela Bastidas-Ramírez, Belinda Vargas-Guerrero, Mayra Mena-Enríquez, Felipe de Jesús Martínez-Limón, Adriana María Salazar-Montes

**Affiliations:** 1 Instituto de Investigación en Enfermedades Crónico-Degenerativas, Centro de Universitario de Ciencias de la Salud, Universidad de Guadalajara, Sierra Mojada 950, Guadalajara 44340, Jalisco, Mexico; esteban.sanchez@alumno.udg.mx (E.A.S.-J.); luz.gasca@academicos.udg.mx (L.E.G.-L.); carmen.gurrola@academicos.udg.mx (C.M.G.-D.); blanca.bastidas@academicos.udg.mx (B.E.B.-R.); belinda.vargas@academicos.udg.mx (B.V.-G.); 2 Instituto de Nutrigenética y Nutrigenómica Traslacional, Centro Universitario de Ciencias de la Salud, Universidad de Guadalajara, Sierra Mojada 950, Guadalajara 44340, Jalisco, Mexico; jose.vcruz@academicos.udg.mx; 3 Centro de Investigación Multidisciplinario en Salud, Centro Universitario de Tonalá, Universidad de Guadalajara, Av. Nuevo Periférico 555, Tonalá 45425, Jalisco, Mexico; luis.hortega@academicos.udg.mx (L.D.H.-O.); guadalupe.mena@academicos.udg.mx (M.M.-E.); 4 Laboratorio Clínico Hospital Civil Nuevo de Guadalajara Juan I. Menchaca, Guadalajara 44340, Jalisco, Mexico; felipelimon99@hotmail.com

**Keywords:** quercetin, nanoparticles, renal injury, renal fibrosis, adenine-induced model, chronic kidney disease

## Abstract

Renal fibrosis is the final stage of chronic kidney injury characterized by glomerulosclerosis and tubulointerstitial fibrosis with parenchymal destruction. Quercetin belongs to the most studied flavonoids with antioxidant, anti-inflammatory, antifibrogenic, and antitumor activity. It modifies the TGF-β/Smad signaling pathway, decreasing profibrogenic expression molecules and inducing the expression of antioxidant, anti-inflammatory, and antifibrogenic molecules. However, quercetin exhibits poor water solubility and low absorption and bioavailability. This limitation was solved by developing a nanoparticles formulation that improves the solubility and bioavailability of several bioactive compounds. Therefore, we aimed to investigate the in vivo antifibrogenic effect of a quercetin nanoparticles formulation. Male C57BL/6 mice were induced into chronic renal failure with 50 mg/kg of adenine for four weeks. The animals were randomly grouped and treated with 25, 50, or 100 mg/kg of quercetin, either macroparticles or nanoparticles formulation. We performed biochemical, histological, and molecular analyses to evaluate and compare the effect of macroparticles versus nanoparticles formulation on kidney damage. Here, we demonstrated that smaller doses of nanoparticles exhibited the same beneficial effect as larger doses of macroparticles on preventing kidney damage. This finding translates into less quercetin consumption reaching the desired therapeutic effect.

## 1. Introduction

Chronic kidney diseases (CKD) are characterized by the progressive and irreversible loss of kidney function with a gradual reduction of glomerular filtration rate (GFR) [1]. CKD health, social and economic burden is a notably important issue in many public and private healthcare systems around the world, including the control of other clinical manifestations of the disease, such as hyperglycemia, hypertension, and dyslipidemia, among others. However, CKD is a “silent” disease without evident clinical symptomatology until all clinical complications are irreversible. Renal fibrosis is the final stage of chronic kidney injury, characterized by glomerulosclerosis and tubulointerstitial fibrosis with parenchymal destruction [2]. In this pathophysiological scenario, the transforming growth factor β1 (TGF-β1) is one of the most critical molecules in fibrosis development. It participates in tissue repair and induces the expression of extracellular matrix proteins such as collagen, elastin, proteoglycans, integrins, and fibronectin [1,2,3]. Although the development of renal fibrosis is well known, there are still no successful pharmacological therapies for its treatment or prevention. Thus, targeting TGF-β1 signaling could be a promissory therapeutic strategy for CKD [4]. In this respect, various bioactive natural compounds have been used as a complementary treatment to prevent and reduce CKD severity. In this sense, flavonoids have been reported to be the most promising [5,6]. The therapeutic action of these compounds has been attributed to antioxidant, anti-inflammatory, antifibrogenic, and antitumoral properties [5,6]. Especially, quercetin is reported as one of the most promising flavonoids. Fruits and vegetables, mainly apples, grapes, tomatoes, and onions, contain considerable concentrations.

Several studies, both in vitro and in vivo, have demonstrated quercetin’s anti-inflammatory and antifibrogenic activity on many chronic health conditions, especially fibrosis. Many biological aspects explain its anti-inflammatory effects, such as a decrease in leukocyte infiltration, iron chelation, inhibition of complement activation, radical scavenging, and myeloperoxidation [6]. Besides, it has been demonstrated that its antifibrotic effect comes from the capacity to act on the TGF-β/Smad and PI3k/Akt profibrogenic pathways, decreasing the expression of proinflammatory molecules as IL-1, IL-6, TNF-α, profibrogenic molecules as TGF-β1, α-SMA, Col1-α1, CTGF, Timp-1, Smad-3, Smad-4, Twist1, Snail, and inducing the expression of antioxidant, anti-inflammatory, and antifibrogenic molecules as IL-10, Smad-6, Smad-7, MMP-9, BMP-7, among others both profibrotic and antifibrotic. Furthermore, quercetin substantially improves the function of the kidney and prevents fibrosis [7,8,9,10]. Additionally, in animal models with hepatic, pulmonary, or dermal fibrosis, quercetin significantly decreased the expression of TGF-β1 and fibroblast activation [7,8,10,11,12].

Although quercetin exerts significant beneficial health effects, the poor water solubility of the molecule limits them, which also impacts its bioavailability. In humans, quercetin bioavailability has been reported at around 24% [13] and 16% in rats [14] if administered orally. Nevertheless, these values depend on the characteristics of the quercetin vehicle or pharmacological presentation [14]. When it is administered in capsules, the bioavailability of quercetin in humans can be as scarce as 1% [15,16]. On the other hand, it has been demonstrated in several bioorganic and inorganic compounds that their nanoparticles formulation considerably increases their solubility and bioavailability [17,18,19,20,21,22]. The present study aims to evaluate if the administration of quercetin nanoparticles improves its antifibrogenic and anti-inflammatory effects compared to their original presentation (macroparticles), with the promising implication of requiring lower doses than that reported in previous works to obtain the same or even better effects.

## 2. Results

### 2.1. Characterization of Quercetin Particles

**Figure 1:**
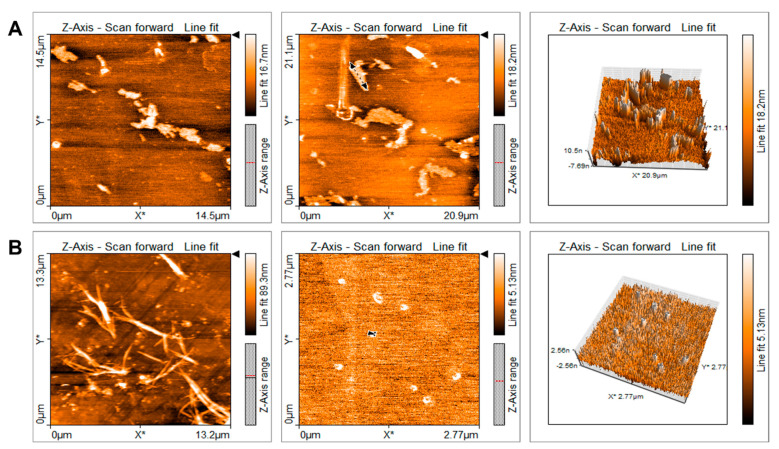
Quercetin nanoparticles characterization, visualization, and comparison with macroparticles. Visualization was made using an atomic force microscope (AFM) and measurement was determined with the Naio software. The average length obtained after three measurements was 35.5 µm for QMPs (**A**) and 140 nm for QNPs (**B**), 253 times less than macroparticles.

Quercetin macroparticles (QMPs) presented irregular shapes with an average size of 35.5 μm (Figure 1A). On the contrary, quercetin nanoparticles (QNPs) prepared by the solvent/antisolvent precipitation method exhibited regular-shaped spheres with a crystal structure with an average particle size of 140 nm. It has been shown that, applying the solvent/antisolvent method, there is a decrease of the size of any particle up to nanometers [23]. The particle size increases the contact surface between solute and solvent, favoring the dissolution [17]. In this work, we were able to decrease the particle size 253 times. Nevertheless, both macroparticles and nanoparticles were diluted in 2% Tween 20 just before administration to animals via oral gavage. With nanoparticles preparation, we observed completed dissolution; in contrast, most of macroparticles were in suspension. Furthermore, a relevant particle size difference of 253 was found between QMPs and QNPs (Figure 1B). Additionally, when QNPs were put in water, they wholly dissolved and stayed in the solution hours after; QMPs, on the other hand, precipitated and did not form a stable solution. Also, QNPs dissolved in a 2% Tween-80 solution, while QMPs formed an emulsion that precipitated minutes after (data not shown).

### 2.2. Effect of Quercetin on Animal Body Weight

**Figure 2:**
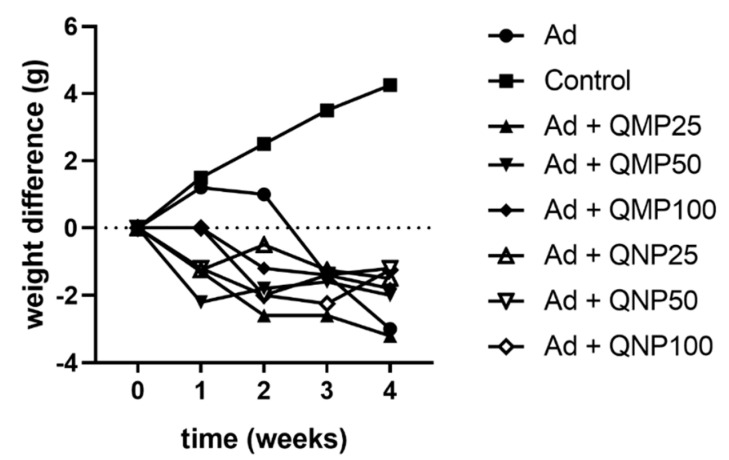
Effect of quercetin macroparticles and nanoparticles on the weight of mice during the 28 day treatment. Bodyweight of mice was registered weekly until sacrifice. QMPs and QNPs treatments prevented weight loss caused by adenine intoxication.

In the control group (Control), the animals gradually increased their body weight from the first week until the fourth week. In contrast, animals intoxicated with adenine (Ad) gained weight in the first week but suddenly decreased their weight until the fourth week. Additionally, animals that received treatment in all doses of either QMPs or QNPs with adenine, at the same time, decreased their weight in the first week, recovering it in the third and fourth weeks. Nevertheless, they did not reach the average body weight of the control group at the final time (Figure 2). Furthermore, the weight of kidneys did not exhibit significant differences among the groups, and there were no differences in the kidney/body weight ratio (data not shown).

### 2.3. Biochemical Markers of Kidney Damage Are Diminished Using Quercetin

**Figure 3:**
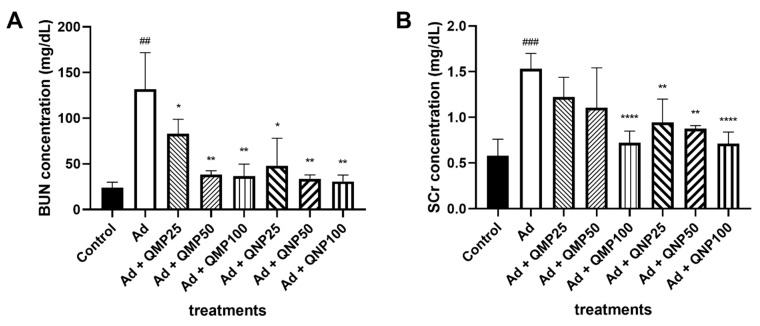
Effect of quercetin macroparticles and nanoparticles on renal markers of CKD. Blood samples from animals of all study groups were centrifuged and analyzed for BUN and SCr concentrations in an automated clinical analyzer. BUN (**A**) and SCr (**B**) concentrations (mg/dL) decreases in both QMPs and QNPs treatments in a dose-dependent manner. QNPs showed a better effect preventing adenine-induced kidney damage than QMPs. Mean ± SD (*n* = 5); ## *p* < 0.01, ### *p* < 0.001 compared to Control group; * *p* < 0.05, ** *p* < 0.01, **** *p* < 0.0001 compared to Ad group. BUN: blood urea nitrogen, SCr: serum creatinine.

Blood urea nitrogen (BUN) and creatinine levels were determined in all serum samples. BUN concentration of 23.94 mg/dL was obtained in the control group, while in the adenine group the levels increased up to 131.81 mg/dL. In addition to this, in the groups treated with QMPs, the levels found were 83.08 mg/dL, 38.29 mg/dL and 36.66 mg/dL for the 25 mg/kg (QMP25), 50 mg/kg (QMP50) and 100 mg/kg (QMP100) doses, respectively. Meanwhile, animals treated with QNPs presented values of 47.69 mg/dL, 33.79 mg/dL and 30.75 mg/dL for the 25 mg/kg (QNP25), 50 mg/kg (QNP50) and 100 mg/kg (QNP100) doses, respectively (Figure 3A). In addition, creatinine serum concentration was 0.58 mg/dL in the control group, and 1.53 mg/dL in the adenine intoxicated group. For the QMP25, QMP50 and QMP100 groups, creatinine serum values were 1.22 mg/dL, 1.11 mg/dL and 0.72 mg/dL, respectively. In this regard, in the QNP25, QNP50 and QNP100 groups, the values found were 0.94 mg/dL, 0.88 mg/dL and 0.71 mg/dL, respectively (Figure 3B).

### 2.4. Quercetin Inhibits Kidney Damage

**Figure 4:**
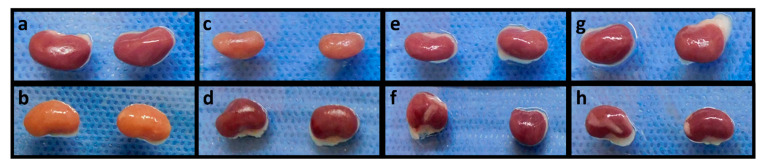
Comparison of kidney damage induced by adenine after 28 days of treatment. Representative photographs of both kidneys were taken. Adenine-intoxicated kidneys show extensive areas of fibrotic tissue (**b**) compared to the control healthy kidneys (**a**). QNPs (**d**,**f**,**h**) prevent the damage in the renal parenchyma to a greater extent than QMPs (**c**,**e**,**g**). (**a**): Control, (**b**): Ad, (**c**): Ad + QMP25, (**d**): Ad + QNP25, (**e**): Ad + QMP50, (**f**): Ad + QNP50, (**g**): Ad + QMP100, (**h**): Ad + QNP100.

After 28 days of treatment, animals were euthanized and samples of both kidneys were taken. The control group showed normal morphology and color of kidney parenchyma (Figure 4a). In contrast, the adenine group exhibited extensive areas of fibrotic tissue (yellowish) (Figure 4b). On the other hand, kidney samples of animals treated with QMPs showed minor areas of fibrotic tissue compared to the adenine group (Figure 4c,e,g). However, the kidney samples treated with QNPs revealed almost a typical morphology of the renal tissues compared with the adenine and QMPs groups (Figure 4d,f,h).

**Figure 5:**
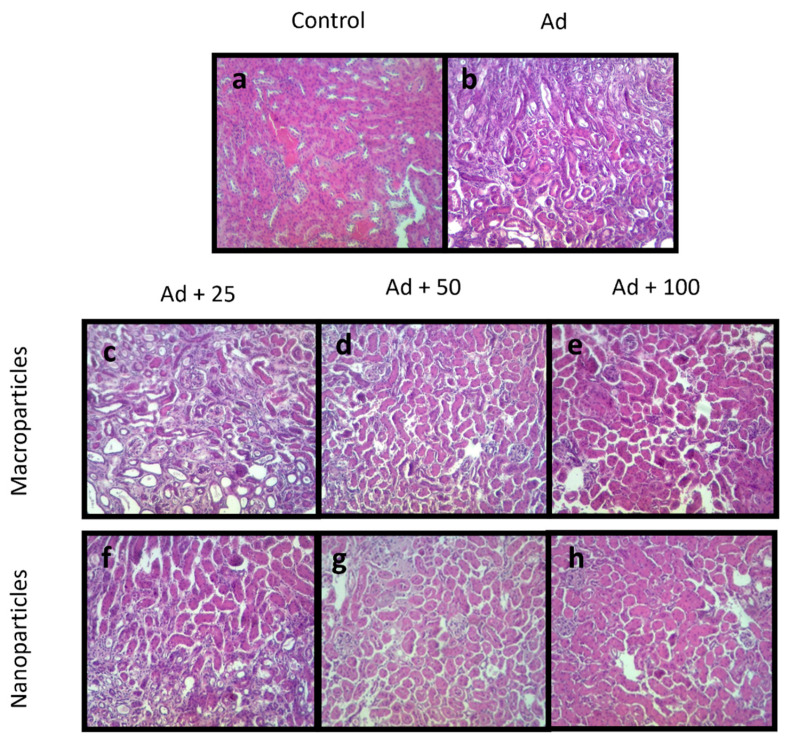
Histopathology analysis of kidney tissue. Kidney samples stained with hematoxylin- eosin were used to analyze inflammation and necrosis. Control kidney samples show normal morphology (**a**), and severe inflammation and necrosis in kidney tissue were observed in adenine-intoxicated animals (**b**). Quercetin treatment prevented the tissue damage induced by adenine where QNPs (**f**–**h**) showed better effect than QMPs (**c**–**e**). (**a**): Control, (**b**): Ad, (**c**): Ad + QMP25, (**d**): Ad + QMP50, (**e**): Ad + QMP100, (**f**): Ad + QNP25, (**g**): Ad + QNP50, (**h**): Ad + QNP100. Magnification of 200×.

Histological kidney sections from all animals were stained with hematoxylin-eosin to analyze the presence and grade of necrosis and inflammation in the tissues after treatments. The control group showed typical glomerular and tubular architecture without necrosis and inflammation (Figure 5a). In contrast, kidney tissues of animals treated with adenine showed dilatation of renal tubules, glomerular damage, and extensive areas of inflammation and necrosis (Figure 5b). Furthermore, the samples treated with QMPs exhibited a significant decrease in tubules dilatation, observing a similar tissue architecture to normal tissue, especially in the QMP100 group, followed by the QMP50 and QMP25 groups (Figure 5c–e). As expected, the kidney damage caused by adenine was minor with QNPs treatment, presenting the best protective effect in the QNP100 group, followed by the QNP50 and QNP25 groups (Figure 5f–h).

### 2.5. Quercetin Prevents Kidney Fibrosis

**Figure 6:**
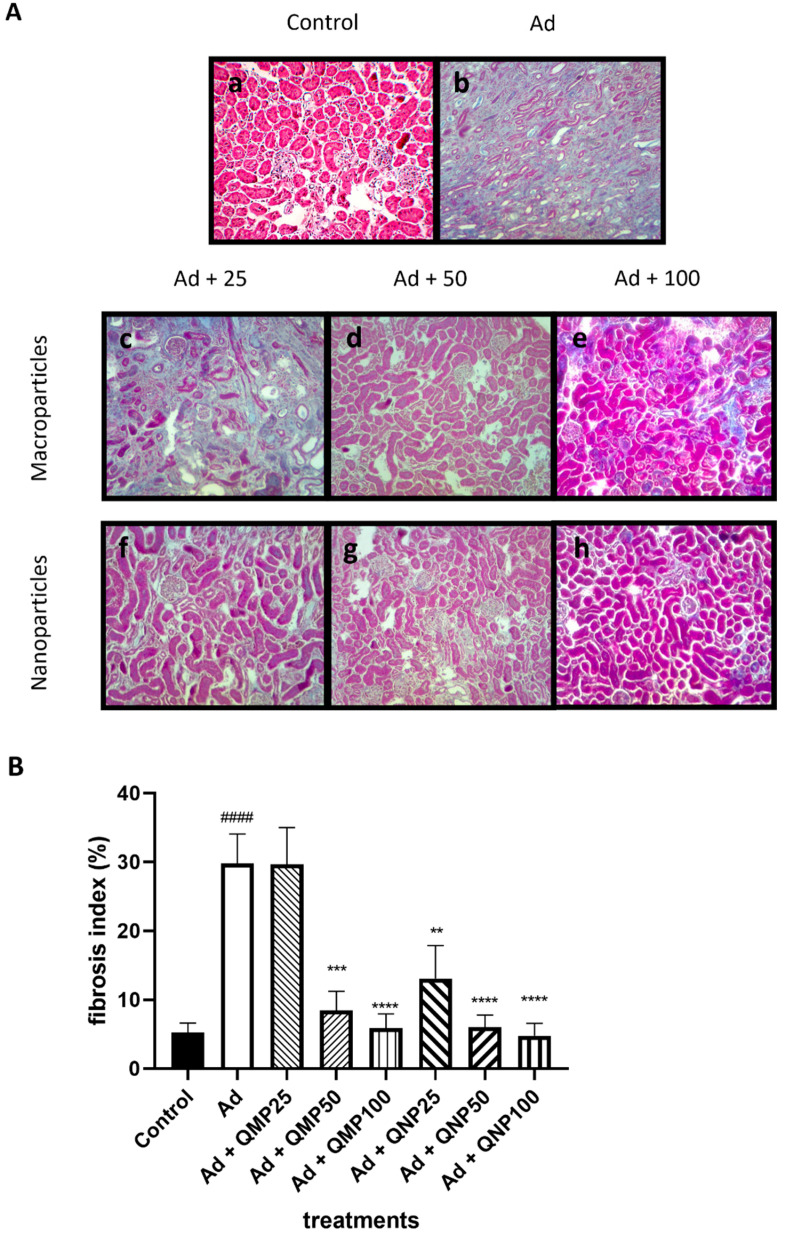
Fibrosis index analysis of kidney tissue. Fibrosis index analysis of kidney tissue was determined by Masson’s trichrome staining. (**A**) Treatment with quercetin diminished the extracellular deposition where QMPs (**c**–**e**) were not as effective as QNPs (**f**–**h**). (**B**) Quercetin diminished the fibrosis index in a dose-dependent manner, showing better results with QNPs than with QMPs. (**a**): Control, (**b**): Ad, (**c**): Ad + QMP25, (**d**): Ad + QMP50, (**e**): Ad + QMP100, (**f**): Ad + QNP25, (**g**): Ad + QNP50, (**h**): Ad + QNP100. Mean ± SD (*n* = 5); #### *p* < 0.0001 compared to the Control group; ** *p* < 0.01, *** *p* < 0.001, **** *p* < 0.0001 compared to the Ad group. Magnification of 200×.

Masson’s trichrome staining evaluated kidney fibrosis in all groups (*n* = 5) to calculate the fibrosis index later. Histological evaluation of the control group displayed a typical architecture with a small deposit of basal extracellular matrix (4.3%) (Figure 6A). The adenine group showed an altered architecture with few nephrons, thick layers of both tubular and glomerular collagen deposition, with 28.73% of fibrosis. On the contrary, the presence of nephrons, thinner fibrosis cords, and a less extracellular matrix content were observed in the quercetin-treated groups. Animals treated with QMPs presented a fibrosis index of 27.9% for the QMP25 group, followed by 8.8% and 5.02% for the QMP50 and QMP100 groups. Likewise, animals treated with QNPs presented a fibrosis index of 12.7% for the QNP25 group, 5.7% for the QNP50 group, and 4.8% for the QNP100 group (Figure 6B).

### 2.6. Quercetin Decreases Profibrogenic and Proinflammatory Cytokine Gene Expression and Increases Antifibrogenic and Anti-Inflammatory Cytokines Gene Expression

**Figure 7:**
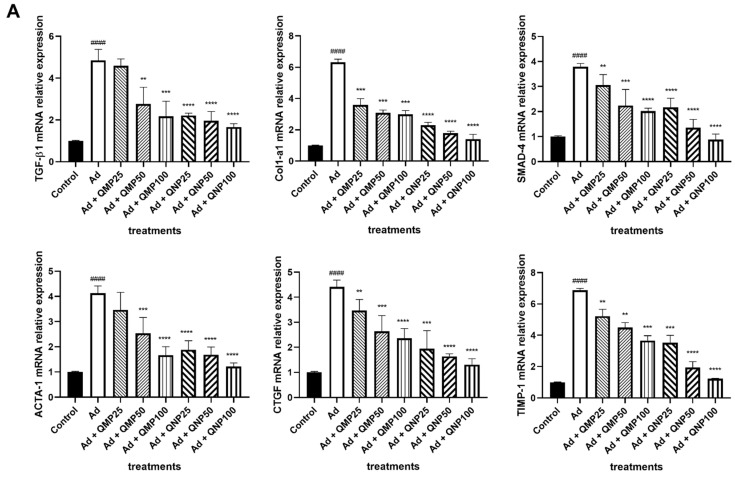

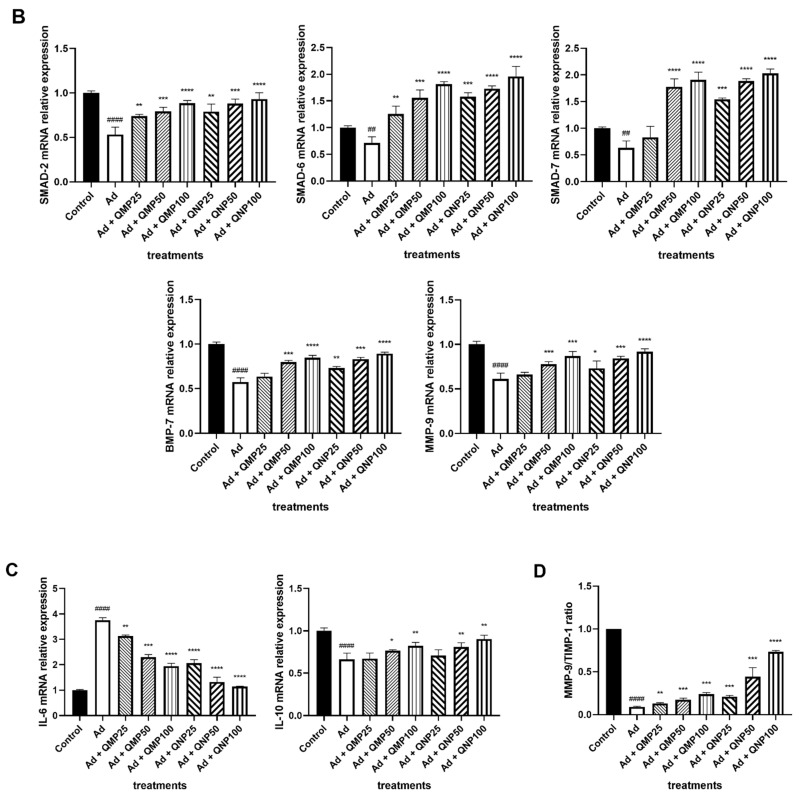
Effect of quercetin macroparticles and nanoparticles on the expression levels of profibrogenic, proinflammatory, antifibrogenic, and anti-inflammatory genes. Expression levels of key genes in the TGF-β/Smad and inflammatory signaling pathways were determined using RT-qPCR. QNPs diminished profibrogenic (**A**) and proinflammatory (**C**) gene expression and induced antifibrogenic (**B**) and anti-inflammatory (**C**) gene expression in a higher extent than QMPs. The MMP-9/TIMP-1 ratio (**D**) increased in the adenine group, while in the groups treated with both QMPs and QNPs, it decreased. Mean ± SD (*n* = 5); ## *p* < 0.01, #### *p* < 0.0001 compared to the Control group; * *p* < 0.05, ** *p* < 0.01, *** *p* < 0.001, **** *p* < 0.0001 compared to the Ad group.

Gene expression levels were determined using the RT-qPCR technique. Analysis of the profibrogenic genes *Col1α1*, *Tgfb1*, *Ctgf*, *Acta1*, *Smad4*, and *Timp1* showed a significant increase in their expression in animals administered with adenine compared to the control group. Interestingly, we observed a dose-dependent decrease in gene expression in all groups treated both with QMPs and QNPs. The best reduction effect was carried out in the group treated with the highest dose of QNPs (Figure 7A).

Regarding the antifibrogenic genes *Smad2*, *Smad6*, *Smad7*, *Bmp7*, and *Mmp9*, a decreased gene expression was observed when the animals were intoxicated with adenine. Nevertheless, the expression was notably increased by the treatment with QMPs and QNPs, having a better effect with QNPs (Figure 7B). Also, the proinflammatory cytokine *Il6* reveals a decrease in its expression levels and the anti-inflammatory cytokine *Il10* exhibits an increase in *Il10* expression with QMPs and QNPs treatments, particularly within the QNP100 group (Figure 7C).

Additionally, the *Mmp9*/*Timp1* ratio was increased in the adenine group compared to the control group and was reduced in the QMPs treatments with a more markable reduction in the QNPs treatments (Figure 7D).

### 2.7. Quercetin Inhibits the Epithelial–Mesenchymal Transition (EMT) and the TGF-β/Smad Fibrogenic Signaling Pathway

**Figure 8:**
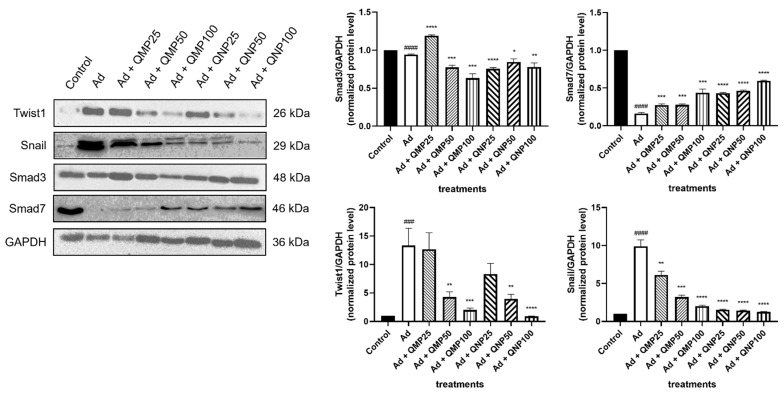
Effect of quercetin macroparticles and nanoparticles on the expression of key proteins involved in the EMT process and the TGF-β/Smad signaling pathway. Expression levels of proteins were determined by Western blotting. Twist1 and Snail decrease their expression in the groups treated with QMPs and especially in the groups treated with QNPs in a dose-dependent manner, while Smad7 increases its expression in the higher dose of QMPs and in all doses of QNPs. EMT: Epithelial–Mesenchymal Transition. Mean ± SD (*n* = 3); ### *p* < 0.001, #### *p* < 0.0001 compared to the Control group; * *p* < 0.05, ** *p* < 0.01, *** *p* < 0.001, **** *p* < 0.0001 compared to the Ad group.

The expression of proteins was carried out by the Western blot technique. The levels of the TWIST1 and SNAIL proteins involved in the epithelial–mesenchymal transition were decreased in a dose-dependent manner in both QMPs and QNPs treatments. However, QNPs in all doses exerted a higher inhibitory effect, especially at the 100 mg/kg dose. In contrast, SMAD7, an inhibitory protein of the TGF-β/Smad signaling pathway, was induced mainly by QNPs treatments, detecting a reduced expression when the animals were treated with QMPs. Finally, SMAD3 levels in QMPs treatments increased in a dose-dependent manner, but, surprisingly, in QNPs treatments did not exhibit such behavior with a higher expression with the 50 mg/kg dose (Figure 8).

## 3. Discussion

In the last years, CKD has increased its incidence in the world population due to co-morbidities like type-2 diabetes, hypertension, and glomerulonephritis, among others. Several in vivo and in vitro studies have described that quercetin exerts anti-inflammatory and antifibrogenic properties in various organs such as the lung, liver, skin, and kidney. As a well-known compound in natural medicine and a safe, natural product [24], quercetin has been used to help in the treatment of many chronic diseases such as obesity and diabetes [25,26,27,28], neurodegenerative diseases such as Alzheimer’s and Parkinson [5,22,29,30], cardiovascular diseases as atherosclerosis [31,32], cancer [33], and other diseases.

There are no available studies of quercetin nanoparticles being used to treat CKD, but only one for acute kidney injury (AKI) [34]. Although, it has been reported in several others, both in vivo and in vitro, that quercetin as macroparticles provides renoprotective, anti-inflammatory, antiapoptotic, and antioxidative effects in the kidney when used to treat diabetic nephropathy [11,27,35], acute kidney injury [36], chronic renal failure [37], chemical toxicity [38], and COVID-19-related acute kidney damage [39]. However, its poor water solubility hinders its low absorption and bioavailability, broadening its clinical utility. Concerning this, works have demonstrated that particles at the nanoscale (10^−9^ m) of different organic and inorganic compounds considerably increase their dissolution and bioavailability [17,18,20,21]. Furthermore, some evidence demonstrated that the pharmaceutical re-formulation of some compounds with low bioavailability into nanoparticles turns them into more bioactive chemical forms due to the increased interaction in the surface area, improving their solubility [19,40]. Additionally, the crystal structure of nanoparticles shows additional features such as increased speed of dissolution and augmented saturation point [41]. Thus, the gain in the solubility and permeability of a drug enhances the oral absorption rate, that is, its bioavailability [42]. 

In this context, a significant improvement in curcumin bioavailability was reported when nanoparticles were compared with macroparticles. Furthermore, the authors highlighted that curcumin nanoparticles increased the time of the molecule in circulation for up to 18 h, while curcumin macroparticles were only detected for 8 h. Notably, a progressive increase in plasma concentration of curcumin nanoparticles was also detected for up to 4 h, which was not exhibited with macroparticles [21]. 

In the same way, other studies, including the anticancer compound 301029, showed an increase in the bioavailability and the concentration in serum when its size was decreased into the nanoscale [17]. Likewise, other compounds, such as carbendazim, a novel antineoplastic drug, also increased their concentration, permeability, and half-life when was administered in the form of nanoparticles, requiring lower doses to obtain the same therapeutic effect [18].

In the present work, we compared the effect of quercetin macroparticles versus quercetin nanoparticles on the prevention of renal fibrosis in an animal model. We used only male animals because it has been reported that fibrosis models in females are challenging to perform and not very reproducible due to female hormonal changes [43,44]. This problem does not happen with males where the times and doses to cause damage are well established and reproducible [45,46]. Specifically, in kidney injury, there are several reports when male rodents are more susceptible to damage by chronic adenine intoxication than females [47,48].

We analyzed several parameters associated with renal damage, such as histological, biochemical, and molecular analysis. In all of them, we could demonstrate a better effect on preventing fibrosis using quercetin nanoparticles than using quercetin macroparticles at similar doses. In this regard, quercetin nanoparticles more effectively inhibit the TGF-β/Smad signaling pathway by promoting the expression of antifibrogenic genes such as *Smad6* and *Smad7*, while inhibiting the expression of profibrogenic genes such as *Col1a1*, *Tgfβ1*, *Smad3*, and *αSMA* (*Acta1*). Additionally, quercetin nanoparticles increased the *Mmp9*/*Timp1* ratio, a critical factor in the remodeling and homeostasis of the renal tissue. Although there is a controversy about the MMP-9 and Timp-1 relationship with kidney damage and CKD, some studies showed a contribution to fibrosis by the higher expression of MMP-9 and lower expression of Timp-1 [49,50], and others revealed contrary observations [51,52]. Our findings align with those reported in several animal models of CKD, particularly regarding tubulointerstitial fibrosis, where MMP-9 increases and TIMP-1 decreases their expressions with quercetin treatments, both with macroparticles and nanoparticles [51,52].

Therefore, these results suggest that quercetin in the form of nanoparticles may improve the absorption in the intestine and its concentration in circulation and bioavailability, facilitating its entry into the cells and activating antifibrogenic and anti-inflammatory signaling pathways. However, one limitation of this study was the impossibility of quantifying the concentration of circulating quercetin due to the sample size and the amount of serum obtained from the animals. Interestingly, complete solubilization of quercetin nanoparticles was observed during the preparation of the quercetin working solution. In contrast, quercetin macroparticles were scarcely dissolved, showing many particles in suspension. Thus, to address the bioavailability question of quercetin nanoparticles, we are performing a new study with the required assays in a rat model, which is a more suitable animal model.

Interestingly, our findings agree with those published by Kakran et al. [20], who stated that modifying the particles size to the nanoscale significantly increases the solubility of organic compounds in aqueous solvents. Moreover, the data presented in our study are in line with those reported by Tousif et al. [21], where the use of curcumin nanoparticles versus macroparticles exhibited better results. Also, our data confirm the observations made by Jia et al. [17,18,19] on the effects of nanonization in the increasing bioavailability properties of poor solubility drugs. Therefore, we confirm that decreasing the size of quercetin increases its therapeutic effect, requiring lower doses of it to obtain the same results. In the same way, a lower dose of quercetin results in the capacity to put nanoparticles as a lyophilized product in capsules or raw powder to be used as a real nutraceutical compound to effectively treat CKD since quercetin, as an isolated macroparticle compound, requires to be taken orally in high doses to be clinically effective in humans due to its scarce solubility properties [53]. Therefore, lower amounts of quercetin would avoid the undesirable adverse effects that have been reported in its prolonged consumption in high doses, such as nausea, headache, and tingling of limbs [54]. In addition to this, in treating kidney damage and some comorbidities such as hypertension and diabetes, chronic pharmacological administration is used for extended periods of time where quercetin can work as an adjuvant, improving the therapeutic effect of these drugs, allowing the recommended doses to be reduced. The doses of quercetin used in this work are based on previous publications where quercetin was tested in different animal models, including one previous report performed by our group (dose used = 100 mg/kg). For this work, we decided to use this dose as the highest to evaluate whether the nanoparticles would have a similar or better effect at a lower dose than a higher one of macroparticles. According to Raegan-Shaw [55], the Food and Drug Administration has suggested that the extrapolation of animal dose to human dose is correctly performed only through normalization to Body Surface Area (BSA), that considers the weight, height, blood volume, plasma protein concentration, oxygen consumption, average caloric expenditure, renal function, and basal metabolism. With these parameters, a Km factor is calculated, 3 for mice and 37 for humans. Then, applying the formula for dose translation, the suggested dose for humans in this case is 567 mg/day, which coincides with that already used in several clinical protocols involving quercetin treatment [24]. However, it has been reported that quercetin in high concentrations could inhibit some drug-metabolizing enzymes such as cytochrome P450 3A subfamily (CYP3A4) [56] and P-glycoprotein (P-gp) [57]. In that sense, its use in lower doses would facilitate drug metabolism, and the global therapeutic effect of both compounds would be more effective.

Moreover, quercetin nanoparticles can positively affect the bioavailability of other drugs, like doxorubicin, a cytotoxic drug used in cancer treatment that has poor oral solubility but increases its bioavailability when CYP3A4 and P-gp are inhibited [58]. In that sense, doxorubicin and quercetin nanoparticles can exert a better anticancer effect than doxorubicin alone. Additionally, the preparation of quercetin nanoparticles by the solvent/antisolvent method, which reduces the size of the molecules to nanometers for its administration, avoids the cytotoxicity effects that has been attributed to the continuous intake of some types of nanoparticles that use delivery vectors as metals (silver, gold, titanium, aluminum, zinc, copper and iron oxides) or non-metals (biopolymers and liposomes) [24,59,60,61].

## 4. Materials and Methods

### 4.1. Reagents

Quercetin and Adenine were obtained from Sigma-Aldrich (St. Louis, MO, USA). TaqMan^®^ probes for real-time PCR were purchased from Applied Biosystems (Foster City, CA, USA). Antibodies for Western blot were obtained from Invitrogen (Rockford, IL, USA).

### 4.2. Quercetin Nanoparticles Preparation

Quercetin nanoparticles were prepared using the method described by Kakran et al. [23]. First, a 5 mg/mL quercetin solution was added dropwise at an 8 mL/min rate to deionized water in a ratio of 1:25 *v*/*v* with constant agitation of 1000 rpm stirrer speed. The quercetin nanoparticles were then concentrated with an evaporator to obtain a 12.5 mg/mL working solution administered later to each animal according to its weight.

### 4.3. Quercetin Particle Imaging and Length Measurement

Quercetin particle imaging was obtained using a NaioAFM atomic force microscope (Nanosurf, Switzerland). We made three different measurements of its length using the Naio software (Nanosurf, Switzerland) and calculated the average particle size.

### 4.4. Animal Model

Forty male C57BL/6 mice with an average weight of 25 ± 2 g were randomly separated in groups of 5 mice and used to develop the kidney fibrosis model. Animals were obtained at five weeks of age from the animal facility of the Juriquilla Campus of the Universidad Nacional Autónoma de México (UNAM) after approval from the Bioethics Council of the University Center for Health Sciences at the Universidad de Guadalajara (Protocol Number: 19–26) and maintained in its animal facilities with food and water ad libitum in a humidity/temperature-controlled room.

Quercetin as either macroparticles or nanoparticles was suspended in 2% Tween 80 and administered to experimental groups at 25, 50, or 100 mg/kg doses two hours before adenine administration. Induction of fibrosis in vivo was made by adenine intoxication, employing 75% glycerin as a vehicle at a dose of 50 mg/kg daily for 28 days via oral gavage using a modified protocol described by Rahman et al. [48]. The control group was administered with vehicle (75% glycerin + 2% Tween 80).

### 4.5. Biochemical Assays

After 28 days of treatment, animals were anesthetized with Zoletil^®^ (tiletamine and zolazepam) (Virbac, Mexico) to obtain blood samples from the retro-orbital sinus at the euthanasia time. Serum was obtained by centrifugation at 3000 rpm for 10 min. Blood levels of urea nitrogen (BUN) and creatinine were measured by a wet method in an automated clinical chemistry analyzer (Beckman Coulter Inc., Brea, CA, USA).

### 4.6. Histopathological Analysis

Both kidneys were obtained after 28 days of treatment, and one section of them was reserved for histopathological analysis. Histological kidney sections of 5 µm thickness were stained with hematoxylin-eosin (HE) and Masson’s trichrome to analyze necrosis and inflammation and evaluate fibrosis percentage, respectively. Histological analysis of necrosis and inflammation of HE slides was performed by a single-blinded certified pathologist. The stained connective tissue/whole evaluated area ratio was calculated in twenty randomly selected fields per slide of Masson’s trichrome slides taken with a VE-B15 optical microscope (Velab Microscopes, Mexico) at a total magnification of 200X, and image processing was made using the Future WinJoe v.1.6 camera control software (Future Optics Sci. & Tech. Co., Hangzhou, China). The fibrosis index was evaluated using the open-source image software CellProfiler™ v.3.1.9 (Broad Institute, USA) [62].

### 4.7. Real-Time Polymerase Chain Reaction (RT-qPCR) Assays

Total RNA was extracted from kidney tissue with Trizol reagent (Invitrogen, USA) according to the modified technique of Chomzynsky and Sachi [63]. Reverse transcription and Polymerase Chain Reaction (RT-PCR) were performed as previously reported [7]. Briefly, the retrotranscription was performed with 2 µg of total RNA in a final volume of 20 µL using the High-Capacity cDNA reverse transcription kit (Applied Biosystems, Foster City, CA, USA). To perform the qPCR analysis 2 µL of cDNA was used. The thermocycler employed was a QuantStudio™ 5 Real-Time PCR System (Applied Biosystems, Foster City, CA, USA). The cycle temperatures and numbers used were according as recommended by the manufacturer. The conditions were the following: Hold 1: 2 min. at 50 °C, Hold 2: 5 min. at 95 °C, Cycling: 45 cycles of 30 s at 95 °C and 40 s at 60 °C. The gene expression of collagen 1 (*Col1a1*), transforming growth factor b1 (*Tgfb1*), connective tissue growth factor (*Ctgf*), tissue inhibitor of metalloproteinases 1 (*Timp1*), interleukin 6 (*Il6*), interleukin 10 (*Il10*), bone morphogenetic protein 7 (*Bmp7*), smooth muscle actin (*Acta1*), matrix metalloproteinase 9 (*Mmp9*), *Smad2*, *Smad4*, *Smad6*, and *Smad7* were quantified with specific TaqMan^®^ probes using the QuantStudio™ 5 thermal-cycler (Applied Biosystems, Bedford, MA, USA). Gene amplification was analyzed by duplicate using glyceraldehyde 3-phosphate dehydrogenase (*Gapdh*) as a housekeeping gene. Data were analyzed using the 2^−∆∆CT^ method [64,65]. The relative expression of every gene was shown as relative expression units.

### 4.8. Western Blot Assays

Western blot analysis of kidney tissue homogenates was performed to analyze SNAIL1, TWIST1, SMAD3, and SMAD7 protein expression (Invitrogen, Waltham, MA, USA). According to our previous real-time PCR analysis, GAPDH (Invitrogen, Waltham, MA, USA) was also used as a housekeeping protein. Proteins were extracted from kidney tissue using P-TER solution (Thermo Fisher Scientific, USA) and quantified by the BCA protein assay kit (Thermo Fisher Scientific, USA). A quantity of 50 µg of total proteins were separated by 10% polyacrylamide gel electrophoresis (SDS-PAGE) under reducing conditions and transferred to nitrocellulose membranes (Thermo Fisher Scientific, Waltham, MA, USA). Blocking was performed using 5% non-fat dry milk in 1X TBST for 1h at 4 °C with constant agitation. Membranes were incubated overnight at 4 °C with primary antibodies diluted 1:1000 (SNAIL1, TWIST1, SMAD3, and GAPDH) and 1:500 (SMAD7) in 1X TBST. Antibody binding was revealed with an HRP-conjugated secondary anti-antibody diluted 1:5000 in 1X TBST using a BM Chemiluminescence kit (Roche Diagnostics, Indianapolis Ind, Indianapolis, IN, USA). Densitometric analysis was performed with a UVP ChemiStudio image analyzer (Analytik Jena, Jena, Germany) using the VisionWorks^®^ software (Analytik Jena, Germany). The semiquantitative analysis of every protein was shown as normalized levels.

### 4.9. Statistical Analysis

Statistical analysis was performed using the GraphPad v.5.0 software for Windows. Shapiro–Wilk test was applied in all analyses to test if the data are normally distributed. Student’s *t*-test was used for data comparison between two unpaired groups or Mann–Whitney U for independent groups when appropriate. Data are presented as the mean ± SD. A *p*-value < 0.05 was considered statistically significant.

## 5. Conclusions

Using the solvent/antisolvent method, we reduced the size of quercetin particles 253 times from the original quercetin macroparticles, obtaining nanoparticles of an average length of 140 nm. As postulated in other studies, we confirmed an improved antifibrogenic effect of quercetin with smaller doses of nanoparticles compared to those obtained with macroparticles. This knowledge opens the gate to further research the promising clinical utility and its integration for the therapeutic and prophylactic management of CKD.

## Data Availability

The data presented in this study are available on request from the corresponding author.
